# Psychological predictors of adherence to lifestyle changes after bariatric surgery: A systematic review

**DOI:** 10.1002/osp4.741

**Published:** 2024-02-24

**Authors:** Jade K. Y. Chan, Lenny R. Vartanian

**Affiliations:** ^1^ School of Psychology UNSW Sydney Sydney New South Wales Australia

**Keywords:** adherence, bariatric surgery, lifestyle change, psychological, psychology

## Abstract

**Objective:**

Adherence to lifestyle changes after bariatric surgery is associated with better health outcomes; however, research suggests that patients struggle to follow post‐operative recommendations. This systematic review aimed to examine psychological factors associated with adherence after bariatric surgery.

**Methods:**

PubMed, PsycInfo, and Embase were searched (from earliest searchable to August 2022) to identify studies that reported on clinically modifiable psychological factors related to adherence after bariatric surgery. Retrieved abstracts (*n* = 891) were screened and coded by two raters.

**Results:**

A total of 32 studies met the inclusion criteria and were included in the narrative synthesis. Appointment attendance and dietary recommendations were the most frequently studied post‐operative instructions. Higher self‐efficacy was consistently predictive of better post‐operative adherence to diet and physical activity, while pre‐operative depressive symptoms were commonly associated with poorer adherence to appointments, diet, and physical activity. Findings were less inconsistent for anxiety and other psychiatric conditions.

**Conclusions:**

This systematic review identified that psychological factors such as mood disorders and patients' beliefs/attitudes are associated with adherence to lifestyle changes after bariatric surgery. These factors can be addressed with psychological interventions; therefore, they are important to consider in patient care after bariatric surgery. Future research should further examine psychological predictors of adherence with the aim of informing interventions to support recommended lifestyle changes.

## 1 INTRODUCTION

Bariatric surgery is among the most effective treatments for obesity, but there is also substantial variability in treatment outcomes.^1^, ^2^ Research suggests that this variability can be attributed, at least in part, to variability in adherence to post‐operative lifestyle changes such as dietary changes, physical activity, and healthcare follow‐up.^3^, ^4^ Despite strong evidence that post‐operative adherence is associated with better weight loss, weight‐loss maintenance, mental health, and quality of life,^5^, ^6^ patients often struggle to consistently enact recommended lifestyle changes. Therefore, it is important to improve the current understanding of the factors that predict post‐operative adherence in the bariatric surgery setting.

Numerous reviews have examined psychological predictors of weight loss outcomes following bariatric surgery, which include (but are not limited to) depressive disorders, substance misuse, and disordered eating^3^, ^6^, ^7^, ^8^ but there has been less of focus on predictors of adherence to post‐operative instructions regarding lifestyle changes. There are two existing systematic reviews of factors associated with post‐operative adherence that focused on a range of demographic (e.g., age and unemployment) and clinical (e.g., pre‐operative BMI) predictors.^5^, ^9^ While knowledge of these factors is valuable in identifying individuals at greater risk of poor post‐operative adherence, they are not modifiable and, thus, can be of limited clinical utility. The review by Hood and colleagues included psychological factors^5^; however, this review contained studies published up until September 2015 and is therefore missing more updated findings.

In order to improve knowledge around factors that can be addressed in clinical practice, the present systematic review aimed to specifically examine clinically modifiable psychosocial predictors of adherence to common treatment instructions after bariatric surgery. For the purposes of this review, the term “psychological” is defined as factors pertaining to psychological wellbeing (e.g., mental health symptoms and disorders, emotional experiences) and cognition (e.g., self‐efficacy, beliefs about adherence). This definition was kept broad in order to capture a wide range of factors that may be of clinical relevance.

## 2 METHOD

### 2.1 Search strategy

PubMed, PsycInfo, and Embase were searched to identify relevant articles from the earliest searchable paper through to August 2022 using keywords and MeSH terms related to two broad concepts: (1) bariatric surgery and (2) patient adherence or compliance. The search terms were based on the search strategies used in existing systematic reviews in the areas of bariatric surgery and treatment adherence, alongside discussions with a research librarian (see Supplementary File for the full search strategy). The search was kept broad in order to capture all studies that measured the relationship between a psychological factor and adherence. Additionally, the reference lists of included articles were hand‐searched to identify relevant papers that were missed in the initial searches.

### 2.2 Study selection

**FIGURE 1:**
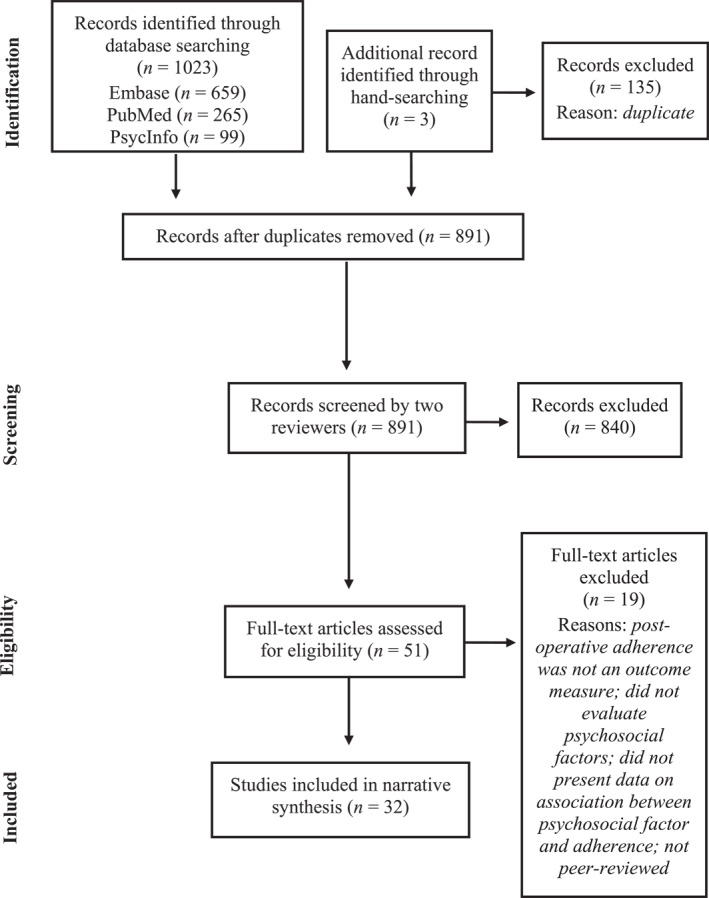
Flow chart of study selection.

A total of 1023 abstracts were located through the database searches and hand‐searching (see Figure 1 for the flowchart of study selection). After removing duplicate results, 891 papers were independently screened by two raters (JC and a research assistant). Inclusion criteria were (1) included patient adherence to instructions after bariatric surgery as an outcome; (2) examined clinically modifiable psychological factors associated with post‐operative adherence; (3) had an adult population (i.e., aged 18 years or older); and (4) written in English. Exclusion criteria were (1) did not have original data (e.g., reviews or editorial pieces); (2) examined only demographic, clinical, or surgical factors associated with adherence; (3) was a case study, trial, or qualitative study, which did not present data on the association between psychological variables and an adherence outcome; and (4) was not peer‐reviewed. Studies were included only if they were considered relevant by both raters and any discrepancies were resolved by a third rater (LV). A total of 32 studies met inclusion criteria and were coded independently by two raters using a coding sheet developed for the purposes of this review.

### 2.3 Data extraction

The following data were coded from the included papers: country where the study was conducted, demographic variables, surgical procedure, time since surgery, and study design. The raters also coded the post‐operative recommendations(s) that were assessed with relation to adherence (e.g., diet, physical activity, follow‐up appointment attendance, support group attendance, and vitamin/supplement use). The selection of these recommendations was guided by the American Society for Metabolic and Bariatric Surgery (ASMBS) Guidelines^10^ and discussions with bariatric clinicians. The psychological factors that were investigated as predictors of adherence in each study were coded as “positively associated”, “negatively associated”, or “not associated”. Although most papers also presented data on demographic and clinical factors associated with adherence, only data related to psychological predictors or correlates of adherence were extracted as these were the outcome measures for this review. In the case of missing data, attempts were made to contact the study's corresponding author via email for further information; however, not all authors responded. Where no response was received, missing data was indicated with “N/A”.

Study quality was assessed using the Joanna Briggs Institute (JBI) Critical Appraisal Checklist for Analytical Cross‐Sectional Studies.^11^ The measure comprises eight items designed to measure the potential risk of bias in study design, conduct, and data analysis. The items cover possible biases in participant recruitment, study sample, validity and reliability of measures used to define exposure, condition, and outcomes, identification/control of confounding factors, and appropriate choice of statistical analysis. The satisfaction of each criterion was rated as “yes”, “no”, “unclear”, or “not applicable” by two independent raters (JC and LV). A study quality rating was given based on the number of criteria rated “yes”. Studies with low ratings were included in the review, but study quality was taken into consideration when synthesizing findings.

### 2.4 Data analysis

Due to the heterogeneity of the included studies, a meta‐analysis was not conducted. A narrative synthesis method was used to summarize the direction of the relationship (positive, negative, or no significant association) between psychological factors measured in the studies and adherence to post‐operative treatment instructions. Studies were grouped by post‐operative recommendations.

## 3 RESULTS

### 3.1 Study characteristics

Among the 32 included studies, the most commonly studied post‐operative instruction was follow‐up appointment attendance (*n* = 17), followed by dietary recommendations (*n* = 11), physical activity (*n* = 6), and support group attendance (*n* = 3). The studies included cross‐sectional designs (*n* = 13), retrospective medical chart reviews (*n* = 10), prospective designs (*n* = 8), and a post‐hoc analysis of randomized controlled trial data (*n* = 1). Psychological factors were assessed using self‐report measures (*n* = 23), by health professionals (*n* = 8), or both (*n* = 1), and adherence was assessed via self‐report (*n* = 14) and medical record data (*n* = 17). One study did not provide information on how adherence was measured. Most studies examined psychological factors pre‐operatively (*n* = 20).

### 3.2 Study quality

The study quality ratings ranged from 3 to 8 (*M* = 6.34), with a maximum score of 8. All studies satisfied the criteria for adequate definition of inclusion criteria. The criterion that was least frequently satisfied was related to strategies to manage confounding factors, with only 50% of studies adequately controlling for confounds in their analyses. Table 1 shows the number of studies that satisfied each of the items in the JBI Critical Appraisal Checklist, and the study quality ratings are included in Table 2.

**TABLE 1:** Numbers and percentages of studies meeting the quality rating criteria of the JBI critical appraisal checklist for analytical cross‐sectional studies.

JBI critical appraisal checklist criterion	Studies satisfying criterion, n (%)
Were the criteria for inclusion in the sample clearly defined?	32 (100%)
Were the study subjects and the setting described in detail?	31 (97%)
Was the exposure measured in a valid and reliable way?	25 (78%)
Were objective, standard criteria used for measurement of the condition?	27 (84%)
Were confounding factors identified?	19 (59%)
Were strategies to deal with confounding factors stated?	16 (50%)
Were the outcomes measured in a valid and reliable way?	22 (69%)
Was appropriate statistical analysis used?	31 (97%)

**TABLE 2:** Study characteristics and psychological factors associated with adherence after bariatric surgery.

Study (Year); Country	Bariatric procedure	Sample size	Sex (%F); Age (M, SD); Pre‐operative BMI (M, SD)	Follow‐up time/time since surgery	Study quality rating (0–8)	Study design	Measure of adherence	Measure of psychological predictor	Psychological predictors (Positive [Pos], Negative [Neg], Non‐significant [NS])
Pre‐operative predictors									
Aarts et al. (2015); The Netherlands	Laparoscopic gastric bypass	105	81%; 45 (9.1) 42.7 (6.1)	6, 12 m	7	Prospective survey study	Single item self‐report of dietary adherence: “I generally/almost/did not follow dietary recommendations”	Mental health history: Single‐item self‐report Hospital anxiety and depression scale Experiences in close relationships ‐ revised	Diet Neg: Mental health history; pre‐operative generalized anxiety symptoms; pre‐operative depressive symptoms; attachment anxiety NS: Attachment avoidance
Barka et al. (2021); France	Sleeve gastrectomy; gastric bypass	92	83.7%; 39.1 (12.1); 44.0 (6.5)	6, 12, 24, 36 m	8	Retrospective chart review	Medical records of follow‐up appointment attendance categorized into frequent attendees (attended at least two follow‐up appointments in a 3‐year period) and non‐attendees (less than two appointments attended)	Medical records of pre‐operative psychological evaluation of psychological and psychiatric history, previous psychiatric therapy, psychological impact of obesity; presence of negative emotions including altered body image, shame, shame facing others, and low self‐esteem	Appointment Neg: Pre‐operative psychological issues related to obesity NS: Presence of negative emotions
Bergh et al. (2016); Norway	RYGB	230	78.3%; 18–60; 44.9 (5.7)	12 m	7	Prospective	Diet: Self‐reported adherence to 6 specific dietary recommendations related to timing of meals, fruit/vegetable intake, low fat meat and dairy, whole grain products, and limiting sugar/fat Physical activity: International physical activity questionnaire (IPAQ)	Motivation: Items from the readiness and motivation interview (RMI) Planning: Single items related to action planning and coping planning General perceived self‐efficacy scale (GSE) Rosenberg's self‐esteem scale (RSES) Body areas satisfaction scale (BASS) Emotion regulation questionnaire (ERQ) Hospital anxiety and depression scale (HADS) The resilience scale for adults (RSA) Relationship assessment scale (RAS)	Diet Pos: Pre‐operative readiness to limit food; self‐esteem; body satisfaction; resilience Neg: Pre‐operative depressive symptoms, night eating NS: Pre‐operative binge‐eating; wellbeing expectations; social competence expectations; planning eating behavior; self‐efficacy; emotion regulation; generalized anxiety; relationship satisfaction Physical activity Pos: Pre‐operative readiness to increase physical activity; planning physical activity; self‐efficacy; self‐esteem; body satisfaction Neg: Pre‐operative depressive symptoms NS: Pre‐operative binge‐eating; night‐eating; wellbeing expectations; social competence expectations; emotion regulation; generalized anxiety symptoms; resilience; relationship satisfaction
Buddeberg‐fischer et al. (2004); Switzerland	Gastric band; gastric bypass; gastric band then bypass	119	76.5% 40 (10.2 44.0 (5.8)	*M* = 10 m	5	Prospective	Self‐reported attendance at follow‐up appointments	Psychosocial stress and symptom questionnaire, which comprised of the HADS, the binge scale questionnaire (BSQ), and the psychosocial assessment questionnaire (PAssQ)	Physical activity Neg: Pre‐operative psychosocial stress in women NS: Pre‐operative psychosocial stress in men
Dixon et al. (2009); Australia	LAGB	227	81% 42.9 (10.4) 44.8 (7.7)	24 m	8	Prospective	Medical records of appointment attendance	Readiness to change: University of Rhode Island change assessment (URICA) scale	Appointment NS: Pre‐operative readiness to change
Gorin et al. (2009); USA	RYGB	196	83.2% 43.6 (10.9) 47.2 (7.4)	6 m	6	Prospective	Diet: Self‐reported frequency of dietary violations that occurred in a typical week (scale: 0; 1–2; 3–6; or >6 times). Physical activity: Self‐reported frequency of engagement in physical activity (scale: <2; 3–5; >5 times per week)	Medical records of pre‐operative psychological evaluation of current and past history of psychiatric conditions. Evaluations were conducted by a clinical psychologist or psychiatric using a scripted interview based on DSM‐IV criteria.	Diet Neg: Pre‐operative mood and eating disorder (combined) Physical activity NS: Pre‐operative mood and eating disorder (combined)
Hecht et al. (2022); USA	RYGB; sleeve gastrectomy	210	84.8% 46.2 (10.2) N/A	12 m	8	Retrospective chart review	Medical records of follow‐up appointment attendance	Health literacy: Rapid estimate of adult literacy in medicine; rapid estimate of adult literacy in medicine—Short form Health numeracy: Brief medical numbers test	Appointment Neg: Pre‐operative limited health literacy; pre‐operative limited health numeracy
Kedestig et al. (2019); Sweden	Laparoscopic gastric bypass	2495	74% 41.7 (10.7) 42.3 (5.0)	2 years	7	Post‐hoc analysis of RCT	Study data on attendance at 2‐year follow‐up appointment	Medical record of pharmacological treatment for depression based on data reported to the scandinavian obesity surgery registry	Appointment Neg: Pre‐operative depression (i.e., received pharmacological treatment for depression)
Khorgami et al. (2015); USA	RYGB	2658	77% 41.2 (12.5) 47.1 (8.1)	1, 3, 6, 12, 18, 24 m	5	Retrospective chart review	Medical records of follow‐up appointment attendance	Medical records of pre‐existing comorbidities	Appointment Neg: Pre‐operative depression
Larjani et al. (2016); **C**anada	RYGB; SG	388	81.2% 44.9 (11.1) 49.4 (8.2)	3, 6, 12, 24 m	8	Prospective	Study data on attendance to postoperative appointments	Assessment of psychiatric history and ongoing psychiatric comorbidities using the mini international neuropsychiatric interview (MINI), conducted by a psychiatrist or psychologist	Appointment NS: Pre‐operative depression; pre‐operative generalized anxiety; psychiatric history
Marek et al. (2015); USA	RYGB	498	72.9% 46.4 (11.6) 47.1 (8.2)	1 year	8	Retrospective chart review	Medical records of attendance at 1‐year post‐operative appointment	Minnesota multiphasic personality inventory‐2‐restructured form (MMPI‐2‐rf)	Appointment Neg: Pre‐operative behavioral/externalizing dysfunction (incl. Antisocial behaviors, history of juvenile conduct problems, substance use, aggressiveness) NS: Pre‐operative emotional/internalizing dysfunction
McVay et al. (2013); USA	RYGB	538	75.3% 45.2 (11.3) 48.5 (8.0)	3 w, 3, 6, 12 m	8	Retrospective chart review	Medical records of 12‐month follow‐up appointment attendance	Depression: Beck depression inventory I (BDI‐I) Psychiatric symptoms: Symptom checklist‐90‐r (SCL‐90‐r) Toronto Alexithymia scale (TAS)	Appointment Neg: Pre‐operative phobic anxiety NS: Pre‐operative depression, hostility, generalized anxiety, interpersonal sensitivity, alexithymia Support group (or behavioral group) Neg: Pre‐operative hostility; generalized anxiety; phobic anxiety NS: Pre‐operative depression; interpersonal sensitivity; alexithymia
Ohta et al. (2022); Japan	LSG	153	60.1% 40.7 (9.6) 45.3 (8.6)	12 m	8	Retrospective cohort study	Medical records of follow‐up appointment attendance	Medical records of psychiatric disorders evaluated by psychiatrists	Appointment NS: Pre‐operative psychiatric disorders
Poole et al. (2005); UK	LAGB	18	88.9%; Range = 25–54 N/A	N/A	5	Retrospective chart review	Appointment: Medical records of follow‐up appointment attendance Diet: Medical record data on compliance to advice related to eating behavior given by two or more health professionals (surgeon, dietitian, or psychiatrist)	Review of psychiatric and surgical case notes for evidence of past or current disordered eating behavior, impulsivity, primary psychiatric diagnoses, and relevant attitudes/beliefs.	Diet Neg: Pre‐operative/history of emotional eating; grazing NS: Pre‐operative/history of binge‐eating disorder; grazing; bulimia; affective mood disorder; alcohol abuse; child sexual abuse; insight; belief that band is responsible for weight loss. Combined diet and appointment Neg: Pre‐operative/history of emotional eating NS: Pre‐operative/history of binge‐eating disorder; bulimia; affective mood disorder; alcohol abuse; child sexual abuse; insight; belief that band is responsible for weight loss.
Sarwer et al. (2008); USA	RYGB	200	82% 43.2 (9.8) 51.4 (9.0)	20 w, 40 w, 66 w, 92 w	6	Prospective	Single‐item self‐report: “How well are you following the diet plan given to you by the dietitian?” on 9‐point likert scale (1 ‐ “not well at all” to 9 ‐ “very well”)	Rosenberg self‐esteem scale Beck depression Inventory‐II Positive and negative affect scale (PANAS) Eating inventory	Diet Pos: Pre‐operative positive affect; cognitive restraint; self‐esteem Neg: Pre‐operative depression symptoms; negative affect NS: Pre‐operative disinhibition
Sockalingam et al. (2013); Canada	RYGB; SG	132	79.5% 43.8 (10.0) 47.6 (7.0)	12m	8	Prospective	Medical records of follow‐up appointment attendance	Beck depression inventory Patient health Questionnaire‐9 (PHQ‐9) Attachment style: Experiences for close relationships (ERC‐16) scale	Appointment Neg: Pre‐operative avoidant attachment NS: Pre‐operative anxious attachment; depression
Toussi et al. (2009); USA	RYGB	112	85% 44.5 (10.9) 48.8 (7.9)	24m	5	Retrospective chart review	Diet: Review of physician consultation notes related to diet behaviors and dietary restrictions Appointment: Medical records of follow‐up appointment attendance	Medical records from a psychological assessment (90‐min semistructured interview) as well as administration of: Eating disorders portion of the structured interview for DSM‐IV Millon behavioral medicine diagnostic test Beck depression inventory II	Diet Neg: History of sexual and/or physical abuse; purging NS: History of mood disorder; eating disorder Appointment Neg: Pre‐operative depressive sx; history of depression; history of psychiatric diagnoses NS: History of eating disorder; history of sexual and/or physical abuse
Vidal et al. (2014); Spain	RYGB; LSG	263	80.6% 44.5 (9.9) 44.9 (5.8)	N/A	5	Cross‐sectional	Medical records of follow‐up appointment attendance	Medical records data, though it is unclear how depression was assessed	Appointment NS: Pre‐operative depression
Wheeler et al. (2008); USA	RYGB; gastric banding	375	84.5% 43.7 (9.5) 46.8 (8.1)	90days	8	Retrospective chart review	Medical records of follow‐up appointment attendance	Beck depression inventory Eating attitudes test	Appointment NS: Pre‐operative depression; maladaptive eating attitudes/habits
Won et al. (2014);USA	RYGB	485	81%46.0 (11.0)47.8 (7.9)	1year, 2years, 3years	6	Retrospective chart review	Medical records of follow‐up appointment attendance	Beck depression inventory II Burns anxiety inventory Yale‐Brown obsessive compulsive scale	Appointment NS: Pre‐operative psychiatric stability (i.e., no psychiatric history or well‐managed symptoms)
*Post‐operative predictors*									
Adler et al. (2018); USA	RYGB	274	95.6% 51 (8.4) 47.4 (8.4)	*M* = *5*.81 year	4	Cross‐sectional	Single item self‐report of dietary adherence: “How well are you following the diet plan given to you by the dietician/nutritionist/surgeon?” (scale from 1 to 9)	Six questions assessing frequency of maladaptive eating behaviors (scale from 1 to 7). Domains included: Grazing/mindless eating; eating foods “off the plan”; after‐dinner hyperphagia; capitulating; loss of control	Diet Neg: Post‐operative grazing; mindless eating; eating foods “off the plan”; over‐eating after dinner; capitulating; loss of control eating
Carmichael et al. (2018); USA	Gastric bypass; gastric band; gastric sleeve; other	320	N/A; N/A; N/A	*M* = *4*.5 years	3	Cross‐sectional	Self‐reported adherence to follow‐up appointments	Participant rating of relationship with primary surgeon and alternate surgeon/non‐surgeon physician (scale from 1 to 5)	Appointment Pos: Post‐operative perceived “good” or “very good” relationship with surgeon
Feig et al. (2019); USA	Gastric bypass; sleeve gastrectomy; other	95	90.5% 49.9 (11.8) 44.2 (7.0)	*M* = *3*.5 years	7	Cross‐sectional	Overall adherence: Bariatric surgery self‐management questionnaire (BSSQ) Physical activity: International physical activity questionnaire (IPAQ)	Positive and negative affect scale (PANAS)—positive affect subscale only Life orientation test revised (LOT‐R) Hospital anxiety and depression scale (HADS)	Overall adherence (diet + physical activity + fluid + supplements) Pos: Post‐operative positive affect; dispositional optimism Physical activity (walking) NS: Post‐operative positive affect; dispositional optimism
Feig et al. (2020); USA	Sleeve gastrectomy; gastric bypass	112	91.1% 50.3 (11.3); 43.9 (7.4)	*M* = *3*.7 years	7	Cross‐sectional	Overall adherence: Bariatric surgery self‐management questionnaire (BSSQ) Physical activity: International physical activity questionnaire (IPAQ)	Weight bias internalization scale—Modified Self‐efficacy for exercise scale Barriers to being active quiz Hospital anxiety & depression scale (HADS)—depression subscale only	Diet Neg: Post‐operative weight bias internalization Physical activity *Moderate‐to‐vigorous physical activity* Neg: Post‐operative weight bias internalization *Walking* NS: Post‐operative weight bias internalization Fluid intake NS: Post‐operative weight bias internalization Supplements Neg: Post‐operative weight bias internalization
Hildebrandt et al. (1998); USA	RYGB	102	88.2% 44.5 (9.7) N/A	*M* = *15*.2 m	3	Cross‐sectional	Self‐reported number of support group sessions attended	Self‐reported mood immediately after surgery and mood at time of study participation	Support group NS: Mood immediately post‐surgery; mood at time of study participation
Hochberg et al. (2015); Australia	LAGB	179	74.9% 49.1 (10.2) 43.05 (7.8)	N/A	6	Cross‐sectional	Medical records of “surgical aftercare” session (i.e., follow‐up appointment) attendance	Questionnaire developed for the purposes of the study, which included a list of 101 commonly perceived barriers to aftercare attendance. Patients rated endorsement of each barrier on a 5‐point likert scale.	Appointment Neg: Post‐operative low motivation to attend follow‐up appointments and/or to lose weight; higher number of post‐operative mental health problems NS: Post‐operative behavioral factors (e.g., “too hard to adhere”); social/family support; unmet expectations
Hunt et al. (2009); USA	N/A	212	78.8% 41.2 (10.5) 49.3 (10.5)	*M* = 572days	6	Cross‐sectional	Godin leisure‐time exercise questionnaire International physical activity questionnaire (IPAQ)	Attitude toward regular exercise: 3 bipolar semantic differential items (7‐point scale)—“useful‐useless”, “wise‐foolish”, “beneficial‐harmful” Affective attitude: 3 bipolar semantic differential items (7‐point scale)—“pleasant‐unpleasant”, “interesting‐boring”, “enjoyable‐unenjoyable” Injunctive and descriptive norms: 3 items rating of agreeance with statements related to exercise social norms on a 7‐point likert scale Perceived behavioral control: 3 items on 7‐point likert scale related to confidence, ease‐difficulty, and control Intention: Single item rating of agreeance with the statement “I intend to exercise regularly over the next 2 weeks” on a 7‐point likert scale	Physical activity Pos: Post‐operative attitudes, subjective norms, and perceived behavioral control (although associations vary by time point and measure of physical activity)
Lester et al. (2014); USA	RYGB	153	100% 51.3 (11.9) N/A	Range = 6–182m	7	Cross‐sectional	The behavior scale, a 10‐item measure using a 4‐point likert scale, which was developed for the study to assess adherence to specific nutrition behaviors over the past month.	Maintenance self‐efficacy: 37‐Item scale developed for the study Recovery self‐efficacy: 39‐Item scale developed for the study Action planning: 4‐Item scale adapted for the study Generalised anxiety disorder assessment (GAD‐7) Depression: Patient health questionnaire (PHQ‐9)	Diet Pos: Post‐operative maintenance self‐efficacy; relapse self‐efficacy; action planning Neg: Post‐operative depression; generalized anxiety NS: Post‐operative coping planning
Miller et al. (2016); Australia	LAGB	183	75.4% 49.2 (10.1) N/A	*Attendees* *M* = *4*.*44*; *Non‐attendees*: *M* = *5*.*14*	6	Cross‐sectional	Medical records of aftercare attendance	Gastric banding aftercare attendance questionnaire, a 31‐item measure developed to assess barriers to aftercare attendance. Patients rated endorsement of each barrier on a 5‐point likert scale.	Appointment Neg: Post‐operative discomfort with attending appointments
Orth et al. (2008); USA	Gastric bypass; LAGB; VBG; revisional surgery	46	N/A N/A N/A	N/A	3	Cross‐sectional	N/A	The support group survey, a survey developed for the study to assess a patient's degree of agreeance with statements related to support group attendance.	Support group NS: Post‐operative beliefs about usefulness, necessity for weight loss, and helpfulness for stress
Raves et al. (2016); USA	RYGB; VSG	298	96.3%; 52.7 (11.9) N/A	*M* = *20*.8 m *(12*.*3)*	8	Cross‐sectional	Single‐item self‐report on how well the individual had followed the dietary recommendations provided by their bariatric dietitian on a 10‐point likert scale Food frequency questionnaire (FFQ), adapted for use with roux‐en‐Y gastric bypass patients Disordered eating after bariatric surgery (DEBS)	Stigma in healthcare: Healthcare weight‐related stigma (HCWS), measure designed for the study by combining the interpersonal sources of weight stigma tool with healthcare‐specific items on the stigmatizing situations inventory Weight bias internalization scale	Diet Neg: Post‐operative weight bias internalization NS: Post‐operative perceived stigma from healthcare professionals
Zhu et al. (2022); China	Sleeve gastrectomy; gastric bypass; other	288	69.1% 33 (N/A) N/A	3m–11year	7	Cross‐sectional	Dietary adherence scale after bariatric surgery (DASBS)	Attitude‐social influence‐efficacy questionnaire after bariatric surgery (ASEQBS)	Diet Pos: Stronger intention; more positive attitude; greater social influence; higher self‐efficacy

Abbreviations: LAGB, laparoscopic adjustable gastric band; RYGB, Roux‐en‐Y Gastric Bypass.

The certainty in this body of evidence may be impacted by the presence of mixed findings for some treatment instructions (although, significant effects are generally in the same direction), the small number of studies available for some post‐operative instructions, the variability in measures of adherence, and variability in study quality.

### 3.3 Psychological predictors of adherence

For appointment attendance, dietary adherence, and physical activity, the studies included in this systematic review were organized into subsections on (1) mental health symptoms and/or disorders and (2) beliefs/cognitions that were associated with adherence to post‐operative instructions. These subsections were not used for the remaining outcomes (support group attendance, adherence to supplement use, and adherence to multiple instructions) because there were relatively few studies included.

### 3.4 Appointment attendance

#### 3.4.1 Mental health factors

Studies investigating psychological factors related to adherence to medical and/or allied health follow‐up appointments have focused primarily on emotional or mental health variables (see Table 2 for summary). In two studies of gastric bypass and sleeve gastrectomy patients, having a history of any psychiatric condition was linked to poorer appointment attendance in the first 24–36 months after surgery.^12^, ^13^ Similarly, laparoscopic adjustable gastric band (LAGB) patients who had not attended any appointments in the previous 12 months were more likely than adherent patients to report that post‐operative mental health problems acted as a barrier to attendance.^14^ However, other studies have failed to find the same association: specifically, pre‐operative experience of “negative emotions”,^12^ pre‐operative psychiatric diagnoses,^15^, ^16^ and psychiatric “stability” (i.e., having either no psychiatric history or well‐managed symptoms)^17^ were not predictive of follow‐up appointment attendance.

Specific mental health disorders have also been investigated in relation to appointment adherence. Pre‐operative diagnosis of depression, high levels of depressive symptomology, and a history of pharmacological treatment of depression were predictive of missing follow‐up appointments in the 24 months after gastric bypass surgery in three separate studies.^13^, ^18^, ^19^ However, four other studies found no link between pre‐operative depression and adherence to follow‐up appointments.^20^, ^21^, ^22^, ^23^ In studies examining anxiety, higher levels of pre‐operative phobic anxiety^20^ and an avoidant attachment style were associated with a lower likelihood of appointment attendance in the first 6–12 months post‐surgery.^21^ However, there was no significant link between adherence and generalized anxiety^15^, ^20^ or an anxious attachment style^21^ in other studies.

Other behavioral and emotional disorders have also been examined in the context of post‐operative adherence. Pre‐operative behavioral problems (e.g., antisocial behaviors and substance use) were associated with poorer follow‐up appointment attendance 1 year after surgery.^24^ In contrast, pre‐operative emotional/internalizing dysfunction,^24^ hostility, interpersonal sensitivity, alexithymia,^20^ and maladaptive eating attitudes and habits^23^ were not significantly related to follow‐up appointment adherence.

#### 3.4.2 Cognitive factors

Patients' beliefs about adherence, about bariatric surgery itself, and about their surgeon have also been studied in relation to appointment attendance. Patients who reported low levels of motivation to attend follow‐up appointments and/or to lose weight after surgery,^14^ and those who felt uncomfortable attending appointments,^25^ were more likely to have missed all follow‐up appointments in the preceding 12 months than were patients who did not endorse those views. Conversely, patients were more likely to attend post‐operative appointments if they reported a “good” or “very good” relationship with their bariatric surgeon compared with those reporting a “poor” or “very poor” relationship.^26^ However, adherence was not significantly correlated with readiness to change before surgery,^27^ nor with beliefs that adherence is too difficult, expectations concerning the outcomes of bariatric surgery, or perceived social/family support.^14^ Having limited health literacy and health numeracy has also been associated with a higher likelihood of missed and “no show” post‐operative appointments, respectively.^28^

### 3.5 Dietary recommendations

#### 3.5.1 Mental health factors

Mental health disorders or symptoms were among the most commonly evaluated psychological factors with regard to dietary adherence. Pre‐operative positive affect, self‐esteem, and body satisfaction were positively linked to dietary adherence. In a prospective study, those who had higher scores on self‐report measures of positive affect and self‐esteem also demonstrated greater improvements in their adherence to dietary recommendations from week 20 to week 92 post‐surgery.^29^ Likewise, a prospective cohort study of 230 patients found that pre‐operative self‐esteem and body satisfaction were positively correlated with dietary adherence 1 year after Roux‐en‐Y Gastric Bypass (RYGB) surgery.^30^

In contrast, pre‐operative depressive symptoms were negatively associated with dietary adherence in two prospective studies.^29^, ^31^ Patients who reported higher levels of depressive symptoms were more likely to identify as being “almost adherent” rather than “generally adherent” at 6 and 12 months after surgery, where the latter category was reflective of better adherence.^31^ Similarly, pre‐operative negative affect predicted less improvement in patients' dietary adherence over time.^29^ Another study also demonstrated a negative correlation between pre‐operative depressive symptoms and dietary adherence, but depressive symptoms were not a significant individual predictor of adherence in a regression model that also included pre‐operative night‐eating, readiness to limit food, and years of dieting experience.^30^

Depressive symptoms assessed *after* surgery also significantly predicted patients' classification as adherent (i.e., adhered approximately/more than half of the time) or non‐adherent (i.e., adhered less than half of the time) to dietary recommendations.^32^ Other studies have found that having a history of mental health help‐seeking,^31^ a history of sexual and/or physical abuse,^13^ and higher levels of pre‐ and post‐operative generalized anxiety symptoms^31^, ^32^ were also negatively predictive of dietary adherence in the 6–24 months after RYGB surgery. Although attachment anxiety was similarly associated with poorer dietary adherence, attachment avoidance did not demonstrate a link to adherence.^31^ Contrary to the findings above, a separate study did not find a significant association between pre‐operative generalized anxiety and dietary adherence.^30^

The literature has also identified eating disorders, disordered eating behaviors, and eating‐related attitudes that are associated with dietary adherence. A history of purging,^13^ pre‐operative emotional eating,^33^ pre‐operative grazing (i.e., picking/nibbling),^33^ pre‐operative night‐eating,^30^ or a history of combined mood and eating disorder^34^ is predictive of poorer adherence to dietary recommendations in RYGB and gastric band patients. Poorer dietary adherence has also been associated with higher frequency of self‐reported grazing, mindless eating, eating foods outside their dietary plan, after‐dinner eating, “capitulating” (i.e., over‐eating following perceived failure in dietary adherence), and loss of control after bariatric surgery.^35^ However, this was reported in a paper with a relatively low study quality rating (4/8). Conversely, patients who, prior to surgery, reported feeling more prepared to limit their food intake demonstrated better adherence after surgery, and those who reported having higher levels of eating‐related cognitive restraint showed greater improvements in adherence over time.^29^, ^30^

#### 3.5.2 Cognitive factors

Beyond these mental health factors, studies have also explored whether patients' cognitions about adherence are related to their dietary adherence behaviors. In a study of 153 female RYGB patients, both post‐operative maintenance self‐efficacy (i.e., confidence in one's ability to adhere to treatment instructions) and post‐operative relapse self‐efficacy (i.e., confidence in one's ability to get back on track after a lapse in adherence) were significant positive predictors of adherence.^32^ In the same study, patients who utilized action planning (i.e., planning details needed to adhere to recommendations) as a coping strategy reported higher levels of dietary adherence compared with those who used this strategy less frequently. However, no significant relationship was identified between coping planning (i.e., planning what to do in the face of barriers to adherence) and dietary adherence. Another study found that, post‐operatively, a stronger intention to adhere, a more positive attitude toward adherence, and higher self‐efficacy regarding adherence were associated with better dietary adherence.^36^

Finally, the role of internalized stigma has also been investigated with relation to dietary adherence. In a study of 298 patients who received RYGB or vertical sleeve gastrectomy within the 5 years prior to participation, those who had higher levels of internalized weight stigma were found to be at increased risk of engaging in disordered eating such as frequent snacking and perceived loss of control over food consumption.^37^ The internalized stigma was also negatively correlated with patients' self‐assessed adherence to post‐operative dietary recommendations. This finding was replicated in a separate study of 112 patients, in which weight bias internalization was associated with poorer self‐reported dietary adherence (but not with adherence to fluid intake recommendations), even when controlling for age, gender, time since surgery, BMI, and surgery type.^38^ However, perceived weight stigma within healthcare settings specifically (e.g., weight‐related discrimination from health professionals) was not associated with dietary adherence.^37^ Conversely, dietary adherence was positively associated with a stronger endorsement of social support or positive social norms related to dietary adherence.^36^

### 3.6 Physical activity

#### 3.6.1 Mental health factors

Five studies evaluated the predictive value of mental health factors (including eating disorders) on patients' exercise adherence.^13^, ^30^, ^34^, ^39^, ^40^ Self‐reported depressive symptoms before surgery were negatively predictive of physical activity in RYGB patients 12 months after surgery.^30^ Similarly, pre‐operative psychosocial stress has been associated with poorer exercise adherence. A study of 119 LAGB and RYGB patients reported that those who were classified as experiencing “great psychosocial stress” (defined as meeting criteria for at least one of: depression, binge‐eating disorder, or psychosocial problems in a self‐report measure) were less likely to engage in regular exercise.^39^ However, this relationship was significant only in female participants.

In a study of RYGB patients, having a history of both mood and eating disorders was associated with a lower likelihood of exercising at least 5 days per week when compared with patients who reported no previous diagnoses, and compared to those who reported only a mood disorder *or* an eating disorder.^34^ That study did not report whether a statistically significant difference in adherence existed between those with just one psychological disorder and those with no disorders. Cross‐sectionally, higher scores on measures of optimism and positive affect were associated with a higher frequency of engaging in moderate‐to‐vigorous physical activity (but not with time spent walking) after bariatric surgery.^40^ However, this relationship was no longer significant once depression and anxiety were controlled for. Bergh and colleagues (2016) also assessed pre‐operative night eating and binge eating in a cohort of RYGB patients and failed to find a significant relationship with physical activity levels 12 months after surgery. Surprisingly, in a separate study, having a history of sexual and/or physical abuse was correlated with *better* exercise adherence in the first 24 months after RYGB surgery.^13^ No significant relationships were found between exercise adherence and measures of pre‐operative anxiety, emotion regulation, resilience, body satisfaction, self‐esteem, or relationship satisfaction.^30^

#### 3.6.2 Cognitive factors

In addition to psychological disorders or mental health factors, patients' beliefs have been linked to exercise adherence as well. This research has focused on beliefs that fall within the framework of the Theory of Planned Behavior (TPB), which encompasses attitudes (i.e., beliefs about the benefits of the behavior), subjective norms, and perceived behavioral control.^41^ The overall model of the TPB was significantly predictive of both intention to exercise and actual leisure‐time physical activity (i.e., physical activity that is not related to work, transportation, or household chores) 6–9 months after surgery, and more than 12 months after surgery.^42^ Out of the three components of the TPB, post‐operative perceived behavioral control (which is similar to self‐efficacy) was the strongest and most consistent predictor of exercise adherence. A separate study also found a significant correlation between *pre‐operative* self‐efficacy and exercise adherence.^30^ Likewise, pre‐operative planning for physical activity (e.g., what exercises to do, when to do them, and how to overcome barriers to exercise) was positively associated with exercise adherence. Conversely, weight bias internalization was negatively associated with time spent engaging in moderate‐to‐vigorous activity (but not time spent walking).^38^ Finally, views that patients reported before surgery, such as readiness to increase physical activity and expectations about wellbeing and social outcomes after surgery, were not significant predictors of exercise adherence.^30^

### 3.7 Support group attendance

Predictors of adherence to support groups or behavioral health groups (more akin to group therapy) were examined in three studies.^20^, ^43^, ^44^ One study examined the relationship between behavioral health group attendance and pre‐operative depression, alexithymia, and psychiatric symptoms (i.e., hostility, anxiety, and interpersonal sensitivity) in a group of RYBG patients.^20^ The behavioral health group utilized a cognitive‐behavioral approach to address issues related to post‐operative psychosocial adjustment, social support, adherence, and mindful eating. That study found that patients who attended fewer than two out of four behavioral groups in the first 12 months after surgery were more likely to have higher levels of pre‐operative hostility, generalized anxiety, and phobic anxiety compared to those who attended three to four groups.^20^ However, these differences were only significant in univariate analyses. When hostility, anxiety, and phobic anxiety were included as predictors alongside travel distance (i.e., between the bariatric clinic and patients' homes) in a multivariate logistical regression, the psychosocial variables were no longer significantly predictive of group attendance. Travel distance remained a significant predictor in the regression, which suggests that it may explain behavioral group attendance over and above psychosocial factors.

Another study of RYGB patients found that self‐reported mood (both pre‐ and post‐operative) as well as emotional or psychosocial problems (operationalized as psychiatric treatment‐seeking and use of psychotropic medications after surgery) were not significantly associated with support group attendance.^43^ In the third study,^44^ researchers investigated the views of patients who underwent bariatric surgery regarding in‐person support group meetings. These views included patients' beliefs about the usefulness, necessity (for weight loss), and helpfulness of support group attendance. No significant differences in views were found between those who had never attended a post‐operative support group and those who had attended at least one group, although non‐attenders were marginally (*p* = 0.07) more likely than attenders to think that support groups would have no effect on weight loss and to think that support groups are not needed post‐surgery. Note, however, that the latter two studies had relatively low ratings (3/8) on the measure of study quality.

### 3.8 Adherence to supplement use

Only one study presented data on adherence to vitamin supplements after bariatric surgery.^38^ In this cross‐sectional survey study of 112 patients after sleeve gastrectomy and gastric bypass, greater weight bias internalization was associated with poorer adherence to recommended supplements.

### 3.9 Adherence to multiple instructions

Three studies measured adherence to a combination of post‐operative instructions. In a small study of LAGB patients (*N* = 18), “non‐compliers” (seven patients who were non‐adherent to dietary recommendations and two patients who failed to attend any follow‐up appointments for at least 12 months) were compared to nine control patients (randomly selected patients who had the same surgeon as the “non‐compliers”).^33^ No differences between the groups were noted with regard to pre‐operative binge eating disorder, bulimia nervosa, mood disorders, alcohol abuse, child sexual abuse history, insight about obesity, and the belief that the band is responsible for weight loss, but non‐compliers were more likely to report pre‐operative emotional eating. Another study examined the relationship between personality traits and affect and found that “overall adherence” (comprising diet, physical activity, fluid intake, and supplement use) was positively associated with post‐operative positive affect and dispositional optimism.^40^ The third study examined adherence to vitamin use alongside other post‐operative recommendations (e.g., dietary instructions, refusing to be weighed) under the category of ‘weight‐loss instructions’,^13^ but no data were presented regarding psychological predictors of adherence to weight‐loss instructions.

## 4 DISCUSSION

Pre‐operative depression/depressive symptoms were commonly associated with poorer adherence to post‐operative instructions, including dietary recommendations,^29^, ^31^ follow‐up appointment attendance,^13^, ^19^ and exercise.^30^ Although six studies did not report a significant relationship between adherence and mood disorders/depressive symptoms, no studies identified a positive correlation between low mood and adherence. There was no discernible pattern of differences between the studies that identified a negative relationship compared to those that did not find a significant relationship in terms of study design, measures used to assess mood, and treatment instruction. Thus, pre‐operative depressive symptoms appear to be a relatively consistent predictor of poorer adherence after bariatric surgery.

Likewise, pre‐operative disordered eating behaviors and attitudes were consistently associated with poorer post‐operative dietary adherence. This highlights the need to thoroughly assess for *and* treat maladaptive eating habits before surgery, particularly as patients may believe, prior to surgery, that their surgery would extinguish unhelpful eating behaviors.^45^ Therefore, patients should be offered education to manage expectations and support to address disordered eating behaviors before and after surgery. Studies have demonstrated that short‐term psychological interventions such as motivational interviewing, cognitive‐behavioral therapy, and acceptance and commitment therapy are effective in improving disordered eating behaviors in patients undergoing bariatric surgery.^46^, ^47^, ^48^

The results for anxiety in its various forms were less clear. Generalized anxiety,^31^, ^32^ phobic anxiety,^20^ and attachment anxiety^31^ were identified as barriers to adherence in some studies, but other studies did not find a significant association between generalized anxiety and post‐operative adherence.^15^, ^20^, ^30^ This variability did not appear to be related to the method used to assess anxiety. Research on the anxiety/adherence relationship in other chronic conditions has similarly found weak associations.^49^ It may be that some forms of anxiety are associated with better or poorer post‐operative adherence while other forms are unrelated, and identifying these relationships would be a worthwhile aim for future research.

When mental health factors were evaluated, it was common for studies to assess pre‐operative symptoms or diagnoses as opposed to post‐operative levels of these variables. Knowledge that pre‐operative depression, for example, predicts post‐operative adherence is undoubtedly useful as it allows the identification of individuals at increased risk of poor adherence. Where appropriate, clinicians could recommend psychological or pharmacological treatments for depression as part of patients' preparation for bariatric surgery to mitigate the risk of suboptimal outcomes. It is equally informative, however, to know whether post‐operative depression similarly impairs adherence. Only one study measured post‐operative depression (using the Patient Health Questionnaire^50^), finding that it was associated with a greater likelihood of being mostly non‐adherent to post‐operative dietary instructions.^32^ This may suggest that there is a need for clinicians to monitor and manage depression post‐operatively to increase adherence and optimize treatment outcomes.

Another important factor to evaluate post‐operatively may be cognitions (i.e., thoughts/beliefs) related to adherence, which was examined in only a few studies. Perceived behavioral control and self‐efficacy after surgery were identified as facilitators of post‐operative adherence in patients who received bariatric surgery.^30^, ^32^, ^36^, ^42^ This finding is in line with studies examining adherence in other chronic conditions.^51^, ^52^ However, beliefs about the usefulness of support groups did not differ between patients who did and did not attend these groups,^44^ although that study classified as “adherent” patients who attended even a single support group and also had a relatively low score (3/8) on the study quality measure. Only two studies investigated the role of internalized weight stigma with consistent findings of a detrimental impact on adherence.^37^, ^38^ Additionally, in the one study that examined the role of health literacy in follow‐up appointment attendance, poorer health literacy was associated with lower attendance.^28^ This is consistent with the literature showing that health literacy positively predicts treatment adherence in other acute and chronic medical conditions and that interventions to improve health literacy can increase adherence.^53^ Therefore, it seems likely that increased health literacy may be beneficial for improving adherence in patients pursuing bariatric surgery, though further research is needed. Given the evidence that cognitions and knowledge play a role in behavior change and adherence,^54^, ^55^, ^56^ there may be of benefit in routinely discussing patients' views about post‐operative adherence in follow‐up appointments and addressing cognitions that may affect adherence. Indeed, psychological interventions aimed at addressing unhelpful cognitions have yielded promising outcomes in patients after bariatric surgery.^57^

Post‐operative predictors of adherence to support group attendance have not been well‐researched, with only three studies identified by this review. No significant relationship was found between support group attendance and psychological factors such as pre‐operative and post‐operative mood, emotional/psychosocial problems, and patients' attitudes toward support groups. Although one study reported negative correlations between support group attendance and pre‐operative hostility and anxiety, these relationships were no longer significant once travel distance was taken into consideration.^20^ With the increased availability and acceptability of videoconferencing options, clinics could overcome the barrier of travel distance by offering online support groups. The literature suggests that over 70% of bariatric patients are interested in remote interventions after surgery,^58^ that telehealth options increase attendance,^59^ and that these interventions are associated with improved eating behaviors and psychological wellbeing.^46^

There is also very little data on psychological predictors of adherence to vitamin/supplement use after surgery.^38^ Although vitamin use may not impact on weight‐loss outcomes directly, there is evidence that patients are at an increased risk of nutritional deficiencies after bariatric surgery^60^ and, thus, research examining psychological predictors of vitamin adherence after bariatric surgery is clearly needed.

The main limitations of this systematic review are that there were few studies available for several of the post‐operative instructions examined, the diversity in how psychological factors and adherence outcomes were assessed, and the variability in study quality ratings.

## 5 CONCLUSION

Post‐operative adherence is important for patients to maximize health benefits after bariatric surgery.^5^, ^6^ However, some patients struggle to consistently follow instructions given by their healthcare team.^13^ The current systematic review identified that pre‐operative depression and disordered eating behaviors are consistently associated with poorer adherence; therefore, it is important to screen for and treat these conditions prior to surgery. Future research should seek to more deeply understand the impact of post‐operative psychological factors, such as depression, anxiety, and cognitions, on adherence, as this could guide psychological management after surgery. The findings of this review also highlight the role of patients' cognitions/beliefs after surgery, as self‐efficacy was consistently associated with better adherence, whereas internalized stigma was associated with poorer adherence. These psychological factors are amenable to treatment and, thus, are key considerations in patient care after bariatric surgery. Moreover, patients report an awareness of the impact of psychological factors on their ability to follow post‐operative instructions, and view psychological care as essential for treatment success.^45^ Therefore, where clinically appropriate, health professionals working with patients pursuing bariatric surgery should offer psychological interventions before and after surgery to build skills that may improve post‐operative adherence and treatment outcomes.

## CONFLICT OF INTEREST STATEMENT

The authors have no conflicts of interest to declare.

## Supporting information

Supporting Information S1
