# Supplementary material for: Psychological predictors of adherence to lifestyle changes after bariatric surgery: A systematic review
Source: Obes Sci Pract. 2024 Feb 24;10(1):e741. doi: 10.1002/osp4.741 (PMC10893879; doi:10.1002/osp4.741)
Supplement: Supplementary file 1 — Supporting Information S1 [file OSP4-10-e741-s001.docx]

**Supplementary File.** Full search strategy

| **Database** | **Search** | **Returns** |
| --- | --- | --- |
| Embase | 1. Keyword (mapped to subj heading & exploded): bariatric surgery   OR  Title for search terms: Bariatric surgery or LAGB or gastric band or gastric bypass or roux-en-y or lapband or biliopancreatic diversion or gastric sleeve or weight loss surgery or weight-loss surgery or sleeve gastrectomy or stomach stapling or duodenal switch or obesity surgery or jejunoileal bypass or duodenal switch or weight reduction surgery or vertical banded gastroplasty or LRYGB or RYGB or BPD-DS or AGB or BPD-RYGB or VBG  AND   1. Keyword (mapped to subj heading & exploded): patient compliance or patient adherence or dietary compliance | 659 |
| PubMed | 1. MeSH term for bariatric surgery OR title/abstract for search terms: (“Bariatric surgery”[Title/Abstract] OR LAGB[Title/Abstract] OR “gastric band”[Title/Abstract] OR “gastric bypass”[Title/Abstract] OR roux-en-y[Title/Abstract] OR lapband[Title/Abstract] OR “biliopancreatic diversion”[Title/Abstract] OR “gastric sleeve”[Title/Abstract] OR “weight loss surgery”[Title/Abstract] OR “weight-loss surgery”[Title/Abstract] OR “sleeve gastrectomy”[Title/Abstract] OR “stomach stapling”[Title/Abstract] OR “duodenal switch”[Title/Abstract] OR “obesity surgery”[Title/Abstract] OR “jejunoileal bypass”[Title/Abstract] OR “duodenal switch”[Title/Abstract] OR “obesity surgery”[Title/Abstract] OR “weight reduction surgery” “vertical banded gastroplasty”[Title/Abstract] OR LRYGB[Title/Abstract] OR RYGB[Title/Abstract] OR BPD-DS[Title/Abstract] OR AGB[Title/Abstract] OR VBG[Title/Abstract])   AND   1. MeSH term for patient adherence, patient compliance, compliance OR adherence, guideline OR patient nonadherence/noncompliance | 265 |

| PsycINFO | 1. Keyword (mapped to subj heading & exploded): bariatric surgery   OR  Title for search terms: Bariatric surgery or LAGB or gastric band or gastric bypass or roux-en-y or lapband or biliopancreatic diversion or gastric sleeve or weight loss surgery or weight-loss surgery or sleeve gastrectomy or stomach stapling or duodenal switch or obesity surgery or jejunoileal bypass or duodenal switch or weight reduction surgery or vertical banded gastroplasty or LRYGB or RYGB or BPD-DS or AGB or BPD-RYGB or VBG  AND   1. Keyword (mapped to subj heading & exploded): patient adherence or treatment compliance or compliance | 99 |
| --- | --- | --- |
| Total | -- | 1023 |

*Note*. MeSH = Medical Subject Heading. All searches were limited to human studies.
